# Chronic Lymphocytic Leukemia

**DOI:** 10.3390/cancers12092504

**Published:** 2020-09-03

**Authors:** Tiziana Vaisitti, Francesca Arruga, Alessandra Ferrajoli

**Affiliations:** 1 Department of Medical Sciences, University of Torino, 10126 Torino, Italy; francesca.arruga@unito.it; 2 Department of Leukemia, The University of Texas MD Anderson Cancer Center, Houston, TX 77030, USA; aferrajo@mdanderson.org

This Special Issue of *Cancers*, made up of nine articles (four original papers, four reviews, and a brief report), is dedicated to chronic lymphocytic leukemia (CLL). CLL is a monoclonal disorder characterized by a progressive accumulation of CD19^+^/CD5^+^/IgM^low^/IgD^low^ mature B lymphocytes and is the most common form of leukemia found in adults in Western countries [1]. The clinical outcome of CLL is quite heterogeneous, with some patients surviving for many years without any therapy and eventually succumbing to unrelated diseases, whereas others die rapidly, within 2–3 years of diagnosis, because of complications from CLL and despite aggressive therapies [2]. In 10–12% of patients, CLL can evolve to a more aggressive lymphoma, typically diffuse large B cell lymphoma, named Richter’s syndrome, which is still a clinical need due to the lack of efficacy of CLL therapies [3,4].

In the past two decades, significant efforts have been made (i) to define prognostic factors to stratify patients and decide best patient management, (ii) to better understand the biology of the disease, and (iii) to identify potential therapeutic targets and validate novel therapeutic opportunities. 

All these efforts have given rise to approximately 12,000 publications exploring the different aspects of the disease. We now have several prognostic factors, in addition to the historical IgVH mutational status [5], that help in stratifying CLL patients and somehow predict disease clinical outcome. These include surface markers (e.g., CD38, CD49d), intracellular kinases (e.g., Zap70), chromosomal aberrations (e.g., del17p, trisomy 12), and genetic variants in critical genes (e.g., *TP53, BIRC3, SF3B1, NOTCH1*) [6,7,8,9,10,11]. When talking about CLL biology, a huge body of work has been made to define how CLL cells signal and which are the main players involved (e.g., B-cell receptor BCR, among others) [12,13,14]; how they communicate with the microenvironment in a bidirectional crosstalk; how they migrate and recirculate from blood to lymphoid organs, where leukemic cells can find the right cocktails of stimuli that lead to proliferation, survival, and most importantly, to resistance to therapy [15,16]. In this context, significant input has come from the use of ad hoc-generated cellular models as well as in vivo studies, based on the use of the TCL1 mouse model and xenografts [17,18,19,20,21,22]. Finally, regarding therapies, we can now rely on a plethora of compounds and therapeutic options that are able to target CLL cells from different sides. Chemotherapy alone or combined with immunotherapy (e.g., Rituximab) remains a gold standard for most patients and in most countries. However, in recent years, a new wave of drugs has reached the market, mostly designed to selectively target the critical pathways that CLL cells rely on (e.g., Ibrutinib, Venetoclax) [23,24,25].

In this issue, some aspects of these three main features of CLL are both reported and discussed as novel findings and reviewed to make an update of the available data. Porrazzo and colleagues present an original article where they describe the prognostic significance of PET/CT applied to CLL to identify patients characterized by a more pronounced rate of proliferating cells in lymph nodes, an inferior outcome, and at higher risk of developing Richter’s syndrome [26]. In a large, retrospective multicenter study, Autore and colleagues report serum lactate dehydrogenase levels as a statistically significant prognostic marker that can predict progression-free survival, treatment-free survival, and overall survival in CLL patients that harbor trisomy 12 [27]. In contrast, Cohen and co-workers reviewed all the main novel biomarkers and their role as prognosticators of disease progression and overall survival in different subsets of CLL patients treated with different therapeutic approaches, including both chemoimmuno- and targeted therapy [28].

Other papers of this Special Issue are instead focused on some aspects of CLL biology and the possibility to selectively target leukemic cells based on their main biological features or how CLL cells behave following targeted therapy administration. Efremov, Turkalj, and Laurenti point their attention to the B cell receptor (BCR), a driver receptor in CLL as well as in other B cell malignancies. They review the main mechanisms that are responsible for its activation, the consequences of BCR pathway stimulation, and how the various BCR inhibitors impact on clinical aspects in CLL [29]. In line with the need to better understand and characterize the functional impact of the available BCR-targeting agents, Kost and colleagues present an original article where they study the effects of idelalisib, a potent PI3Kδ inhibitor, administered alone or in association with bendamustine. Their data indicate a synergism between these drugs, even in the more aggressive subset of patients or in the presence of a protective microenvironment. Moreover, they report a global RNA synthesis decrease in CLL cells treated with idelalisib, suggesting that this drug may be effective by downmodulating several critical pathways for CLL cell survival [30]. Patrussi, Capitani, and Baldari present an overview of the functions of an adaptor protein, p66Shc, known to have proapoptotic and pro-oxidant roles. They discuss how this protein controls different aspects of B cells biology, including cell trafficking, and the peculiar p66Shc deficiency that characterizes CLL cells and the biological meaning of this loss [31]. 

Andreani and colleagues present the state-of-the-art on tumor suppressor genes and proteins in CLL, a field still largely unexplored. They describe how tumor suppressors impact on survival and apoptosis of leukemic cells and finally, discuss how these proteins may represent novel opportunities to target these tumor cells [32]. Another field in CLL that is still poorly covered is metabolism of leukemic cells. In the research article presented by Chowdhury et al., they measured the mitochondrial respiration capacity of CLL cells and compared this rate to normal B lymphocytes, finding that tumor cells are characterized by a higher respiration rate and that this feature is correlated to several known negative prognosticators in CLL. More interestingly, they showed the mitochondrial respiration of leukemic cells drop-off in Ibrutinib-treated patients, suggesting that this drug is capable of impacting CLL mitochondrial bioenergetics [33]. 

Finally, this Special Issue includes a brief report by Koch and colleagues that summarizes the results from a meta-analysis on a large cohort of TCL1 mice, showing that leukemia progression is significantly accelerated in female TCL1 mice compared to males and that additional genetic lesions, besides the TCL1 transgene, further contribute to disease progression [34]. These findings pose several questions on the use of this model, the only one, up to now, available to mimic the human disease apart from xenografts. They also suggest that the experimental design needs to be carefully planned, taking in mind these potential confounding elements.

Overall, this Special Issue of *Cancers* is a collection of articles discussing different aspects of CLL, some of which are in an advanced stage of knowledge, while others are just at the beginning, opening for novel and interesting points of discussion in the near future. Moreover, this issue underlines our need to understand tumor biology to design novel and more efficacious therapies to eradicate this leukemia.
